# Choroidal Assessment in Patients with Type 2 Diabetes Mellitus and Non-Proliferative Diabetic Retinopathy by Swept-Source Ocular Coherence Tomography and Image Binarization

**DOI:** 10.3390/medicina58070918

**Published:** 2022-07-10

**Authors:** Otilia Obadă, Anca Delia Pantalon, Gabriela Rusu-Zota, Anca Hăisan, Smaranda Ioana Lupuşoru, Dorin Chiseliţă

**Affiliations:** 1 Department of Ophthalmology, “Grigore T. Popa” University of Medicine and Pharmacy, 16 Universităţii Street, 700115 Iaşi, Romania; chiselita.dorin@gmail.com; 2 Department of Ophthalmology, “Saint Spiridon” Clinical Emergency Hospital, 1 Independenţei Street, 700111 Iaşi, Romania; 3 Department of Pharmacology, “Grigore T. Popa” University of Medicine and Pharmacy, 16 Universităţii Street, 700115 Iaşi, Romania; rusu.i.gabriela@umfiasi.ro; 4 Department of Emergency Medicine, “Grigore T. Popa” University of Medicine and Pharmacy,16 Universităţii Street, 700115 Iaşi, Romania; anca.haisan@umfiasi.ro; 5 Department of Surgery, “Grigore T. Popa” University of Medicine and Pharmacy, 16 Universităţii Street, 700115 Iaşi, Romania; smaranda-ioana.lupusoru@d.umfiasi.ro; 6 Oftaprof Ophthalmology Clinic, 54 Stejar Street, 700327 Iaşi, Romania

**Keywords:** choroidal assessment, non-proliferative diabetic retinopathy, swept-source ocular coherence tomography, image binarization

## Abstract

*Background and Objectives:* The aim of this study was to evaluate choroidal structure and vascularity indices in patients with non-proliferative diabetic retinopathy (NPDR). *Materials and Methods*: Sixty-three eyes from sixty-three patients were evaluated: 21 from healthy subjects, 20 with diabetes mellitus (DM) and no diabetic retinopathy (DR), and 22 with DM and non-proliferative diabetic retinopathy without diabetic macular edema (DME). Each patient underwent ocular examination, macular swept-source ocular coherence tomography (SS-OCT) imaging, glycemic control, and systemic high blood pressure (HBP) evaluation. Subfoveal choroidal thickness (SF-CT) was manually assessed on a line scan. Line scan OCT images were exported to ImageJ program. The areas under a 1.5, 3 and 6 mm horizontal line centered on the fovea were assessed by converting the OCT images to binary images, and total choroidal area (TCA), luminal area (LA), stromal area (SA), LA:SA ratio, and choroidal vascularity index (CVI) were evaluated. SF-CT and choroidal parameters were compared between groups, and correlations with ocular and systemic factors were analyzed. *Results*: SF-CT, TCA, LA, and SA were similar between groups. CVIs were significantly different between groups for all three studied areas (CVI-1.5: 66.21% vs. 66.06% vs. 63.74%, *p* = 0.003; CVI-3: 65.88% vs. 66.46% vs. 63.79%, *p* = 0.008; CVI-6: 64.79% vs. 65.40% vs. 63.61%, *p* = 0.032). NPDR patients had significantly lower CVIs compared to DM patients (*p* < 0.05). No association of choroidal parameters with glycemic control, DM duration and HBP was found significant (*p* < 0.05). *Conclusions*: Choroidal assessment by SS-OCT and image binarization in healthy subjects, subjects with DM without DR, and subjects with DM and NPDR indicated that CVI changes were identifiable and significant in early DR. The lack of association with ocular and systemic factors suggest that CVIs are reliable assessment parameters of choroidal vascular structure.

## 1. Introduction

Diabetic retinopathy (DR) is a common ocular complication of diabetes mellitus (DM) that can lead to impaired visual function and permanent visual loss in advanced stages of disease [1].

Choroidal disorders in diabetes are referred to as diabetic choroidopathy (DC), an entity described by Hidayat and Fine who identified choriocapillaris alterations, tortuosities of the medium and large vessels, narrowing of the vascular lumen, microaneurysms and thickening of the basement membrane [2]. In DC, there is first a reduction of the choriocapillaris and then a loss of them. Choriocapillaris compromise in DC may be diffuse with segmental alterations, but without complete absence, and focal with complete absence over a well-defined area of choriocapillaris. Focal loss of choriocapillaris is four times more important in diabetic patients compared to elderly nondiabetic patients [3].

The identified risk factors for the development of DC are DR, DM control and treatment regimen and systemic blood pressure control [4,5,6]. Pathological evidence of DC suggests that choroidal vasculopathy in DM may play a key role in the pathophysiology of DR, given the organization of the ocular vascularization, in which the external retina is nutritionally dependent on the proper functioning of the choroid [7]. Choriocapillaris compromise induces hypoxia in the retinal pigment epithelium (RPE) and external retina. A hypoxic RPE induces VEGF production and stimulates angiogenesis [8,9]. Pathology studies have shown that the loss of choriocapillaris is associated with the development of choroidal neovascularization at that level, in or above the Bruch’s membrane [6].

Studies have shown that the choroid and several systemic elements are related. The choroidal blood flow is under neural control. Parasympathetic innervation is responsible for vasodilatation and increases the choroidal blood flow. Conversely, sympathetic innervation is responsible for vasoconstriction and decreases the choroidal blood flow. Aging and various vascular ocular and/or systemic diseases contribute to neural control dysfunctions with respect to choroidal blood flow and further amplifies the retinal dysfunctions [10]. Sympathetic control is a mechanism of protection against acute HBP. Altered vascular elastic properties are found both in the choroidal and intrarenal vascular system in hypertensive patients [11]. Few data are available with respect to the influence of hypercholesterolemia on the choroid, although it has been hypothesized that the choroid can be affected by atherosclerotic alterations and can increase its thickness in hypercholesterolemic status [12,13]. DM impacts choroidal vascularization, but studies have shown contradictory results with respect to choroidal thickness (CT) in this altered metabolic status [13].

Advances in ocular imaging have improved the ability to visualize the choroid, and studies on choroidal vessels suggest that DC may be directly involved in the pathogenesis of DR [14]. In vivo imaging of DC can be performed by various methods. Indocyanine green angiography was the first imaging method to identify DC findings as early hypofluorescence and late hyperfluorescent and choroidal nonperfusion areas [15]. Laser Doppler flowmetry imaging allowed for the quantification of choroidal blood flow in diabetic patients but only at the subfoveal level where the retinal vascularization does not overlap [16]. In proliferative diabetic retinopathy (PDR), both the subfoveal flow and choroidal volume are reduced, changes also observed to varying degrees in patients with non-proliferative diabetic retinopathy (NPDR) compared to control subjects [17].

Optical coherence tomography (OCT) imaging is important in non-invasive monitoring of DR progression. Choroidal assessment can be performed by the spectral-domain OCT and swept-source (SS) OCT technologies. The longer wavelength of SS-OCT (1050 nm) compared to SD-OCT (840 nm) has the advantage of reducing the dispersion caused by RPE, thus ensuring a better view of the choroid with a more accurate identification of both Sattler and Haller layers as well as the choroidal–scleral junction (CSJ) [18,19]. The ability to render high-resolution images using the two types of OCTs allowed the choroid to be evaluated quantitatively.

Several studies have evaluated the CT at different stages of retinal damage. The results highlighted the differences and brought CT assessment into a controversial area due to the highly variable outcomes [20,21]. The study of the choroidal vascular parameters obtained by binarizing of the images acquired by OCT can bring new insights regarding the pathogenesis of DR and DC. Images binarization is a useful method because it allows for the study of the structures that make up the choroid by converting a grayscale image (0–256 grayscale) into a white (1) and black (0) image. Image binarization also has limitations, as black pixels are thought to represent vascular areas and white pixels choroidal stromal areas [22,23]. The choroidal vascularity index (CVI) indicates the proportion of vascular choroid and is calculated as the ratio of luminal (vascular) area (LA) to total choroidal area (TCA). CVI is considered a more stable marker for choroid evaluation [24,25].

Most studies have focused on assessing subfoveal choroidal features. There are few studies that extend the image processing beyond the subfoveal area. The aim of our study was to assess the choroid in patients with diabetes without DR and in patients with diabetes and NPDR by analyzing the choroidal area both subfoveal and extrafoveal, to identify if there are differences between the measured areas and if the choroidal changes are correlated with demographic, ocular, and systemic factors.

## 2. Materials and Methods

### 2.1. Study Subjects

This comparative cross-sectional study included 63 eyes from 63 patients evaluated in the Department of Ophthalmology within the “Saint Spiridon” Clinical Emergency Hospital, Iaşi, Romania, between August 2017–February 2018. The study was conducted in accordance with the Declaration of Helsinki, and the protocol was approved by the Ethics Committee of “Grigore T. Popa” University of Medicine and Pharmacy (no. 13948/13 July 2017). All subjects signed an informed consent upon inclusion in the study.

The patients were grouped into 3 categories: (1) 21 eyes from 21 healthy patients (group 1), (2) 20 eyes from 20 patients with DM without DR (group 2) and (3) 22 eyes from 22 patients with DM and NPDR without diabetic macular edema (DME) (group 3).

All patients underwent a complete ophthalmological examination that included best corrected visual acuity (BCVA), spherical equivalent (SE), intraocular pressure by Goldmann tonometer, anterior segment biomicroscopy and dilated fundus examination. Lens status (phakic/pseudofakic) was recorded, and axial length was measured by contact ultrasound ocular biometry (ALCON Ultra Scan Imaging System) by an experienced investigator (O.O.). After mydriasis with Tropicamide 1%, SS-OCT images were obtained.

Demographic data (age, sex) were recorded. Fasting blood sugar (mmol/L), glycated hemoglobin (%) and the presence (yes/no) of controlled high blood pressure (HBP) were recorded in all 63 patients. Duration of diabetes (years) and anti-diabetic treatment were recorded for diabetic patients. We included only patients with type 2 DM.

Patients with history of trauma, previous ocular surgeries, glaucoma, intraocular hypertension, uveitis, age-related degeneration, retinal vascular occlusions, systemic autoimmune or inflammatory diseases, and uncontrolled HBP were excluded.

### 2.2. Image Acquisition

The subjects were imaged with DRI Triton SS-OCT (Topcon Corporation, Tokyo, Japan) between 1 p.m. and 3 p.m. to avoid diurnal variation of the choroid [26,27]. Two SS-OCT scans were performed: a 3D horizontal macular cube scan (7.0 × 7.0–512 × 256) and a line scan (9 mm–1024 × 992) centered on the fovea. The first scan was performed for structural analysis of the macula, and the second scan was employed for choroidal assessment. On the line scan, we evaluated the posterior choroidal boundary to identify the CSJ. For each patient, we recorded whether the CSJ had been accurately identified (yes/no). In addition, we evaluated the presence (yes/no) of the suprachoroidal layer (SCL) identified as a hyporeflective band compared to the choroidal stroma [28,29,30]. CSJ was defined as the inner border of the sclera when SCL was absent and the inner border of the SCL when the SCL was present. SCL was not included in the measurements of the subfoveal choroidal thickness (SF-CT).

SF-CT was defined as the perpendicular between the hyperreflective line of Bruch’s membrane and the CSJ. SF-CT was measured manually once by a single experienced investigator (O.O) using the in-built calipers tool (IMAGEnet software). Subfoveal retinal thickness (SF-RT) was defined as the perpendicular between the internal limiting membrane and Bruch’s membrane. SF-RT was measured automatically once.

Only high-quality images with quality index (QI) above 90 were included in the study.

### 2.3. Image Binarization

The OCT line scan images were exported as current B-scan (original). Image binarization used a free software, ImageJ (https://imagej.nih.gov/ij/) (accessed on: 20 November 2020) [31], and followed the main protocol described by Sonoda and Agrawal [23,24,25,32].

The following steps were undertaken for image analysis: (1) the image was opened in ImageJ; (2) the image scale was set; (3) using the ”straight” in-built tool, a 1.5 mm horizontal line centered on the fovea was drawn; (4) using the ”polygon selections” in-built tool, we delineated the total choroidal area (TCA) beneath the 1.5 mm horizontal line between the Bruch’s membrane and CSJ as defined previously; the selected polygon was the region of interest (ROI); SCL was not included in the ROI; (5) using the function ”add-[t]”, the ROI was added to ROI manager window; (6) the image was converted to an 8 bit image; (7) auto local threshold by Niblack method and radius 15 was used for image binarization; (8) the binarized image was evaluated at the posterior choroidal boundary, and when the CSJ definition was not respected, the posterior selection was adjusted for no SCL be included in the ROI and no stromal area be excluded from the ROI; (9) the new ROI was added to ROI manager window, and the first ROI was deleted; (10) the binarized image was converted to an RGB (Red Green Blue) image; (11) ”color threshold” was applied, and the brightness was adjusted by setting the first bar to 0 and the second to 254 for highlighting the area of vascularity; (12) the image was added to ROI manager; (13) the areas from ROI manager were selected, and the ”AND” function was applied to merge the two previous areas; (14) the third area represents the luminal area (LA) (dark pixels) and was added to ROI manager; (15) the first area (TCA) and the third area (LA) were then measured using ”measure” in-built tool. The stromal area (SA) and the choroidal vascularity index (CVI) were calculated from TCA and LA. SA (light pixels) was obtained by subtracting LA from TCA. CVI was defined as LA/TCA. TCA, LA, and SA were expressed in mm^2^ and CVI in percentages (%).

**Figure 1:**
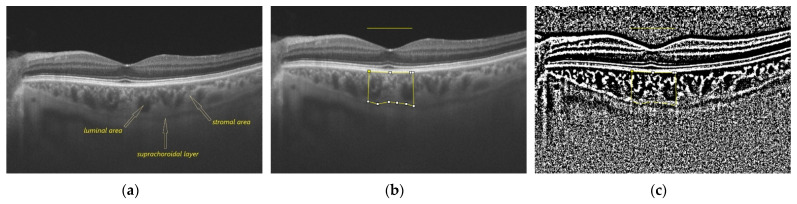

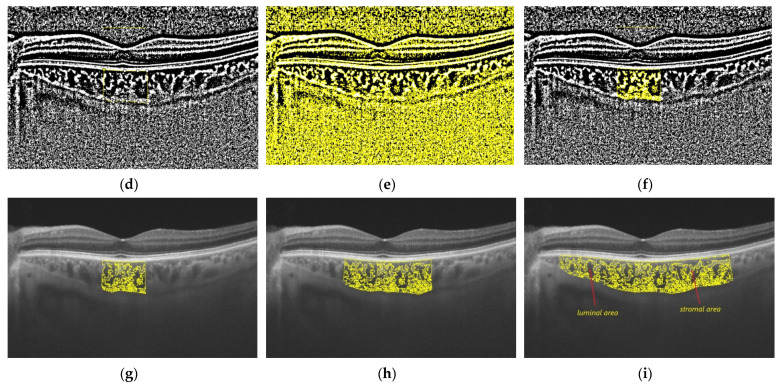
Illustration of image binarization protocol: (**a**) SS-OCT image of a patient with DM without DR; the suprachoroidal layer, luminal area, and stromal area are indicated by arrows; (**b**) a polygonal selection of the choroid under a 1.5 mm horizontal line centered on the fovea; (**c**) image binarization by Niblack method; (**d**) small adjustments of the polygon points after a better visualization of the choroidal-scleral junction; (**e**) color thresholding to highlight the vascular area; (**f**) luminal area; (**g**) final result after overlaying the luminal area measured for the area under the 1.5 mm horizontal line centered on the fovea on the SS-OCT image; (**h**) representation of the final result after applying the same protocol for the area under the 3 mm horizontal line centered on the fovea; (**i**) representation of the final result after applying the same protocol for the area under the 6 mm horizontal line centered on the fovea.

The measured choroidal parameters were TCA and LA; the calculated choroidal parameters were SA, LA/SA and CVI. The choroidal parameters were assessed for each subject for the area under a 1.5, 3 and 6 mm horizontal line centered on the fovea. The above protocol was applied twice by the same experienced investigator (O.O.) two days apart. Figure 1 illustrates the above-mentioned protocol of image processing.

### 2.4. Statistical Analysis

Statistical analysis in our study used SPSS^®^ 28 (Armonk, NY, USA: IBM Corp). Data were recorded as mean ± SD (standard deviation). The BCVA values were converted from decimal visual acuity to the logarithm of the minimum angle of resolution (logMAR). Intra-rater reliability was measured by intraclass correlation coefficient (ICC) using the absolute agreement model. Shapiro–Wilk test was used for testing the normality. Pearson Chi-square test and Fisher exact test were used for differences between categorical variables. Mann–Whitney U test was used for differences in duration of DM and QI. One-way analysis of variance test (ANOVA) was used for differences between groups when the parameters were normally distributed; if the test was significant, a Bonferroni post hoc analysis was performed for pairwise comparisons. Kruskal–Wallis test was used for differences between groups when the parameters were non-normally distributed. Pearson and Spearman correlation test were used for testing correlations. Multiple regression analysis was further employed when correlations between variables was statistically significant. The strength of the effect of each individual independent variable to the dependent variable was evaluated by beta coefficient (*β*). A *p* value less than 0.05 was considered statistically significant.

## 3. Results

### 3.1. Characteristics of the Participants

Demographics, ocular, and biochemical characteristics of the three groups are presented in Table 1.

Patients in group 3 were significantly younger than those in group 1 (*p* = 0.01) and group 2 (*p* = 0.006). Sex distribution was similar between groups. There were no statistically significant differences with respect to BCVA, SE and axial length (*p >* 0.05). Patients in group 3 had significantly higher IOP compared to group 1 (*p* = 0.001) and group 2 (*p* = 0.025). Over 90% patients in group 3 were phakic, while approximately half patients in group 1 and group 2 were pseudophakic.

Only 5% patients were treated with both OADs and insulin in group 2, compared to 41% in group 3. DM duration did not differ significantly in group 2 and group 3. HBP presence was similar between all three groups. Fasting blood sugar was significantly different between all three groups (*p* < 0.05), while HbA1c values were different only between group 1 and group 2 (*p* = 0.003), and group 1 and group 3 (*p* = 0.000), respectively.

### 3.2. Choroidal and Retinal Assessment

#### 3.2.1. SS-OCT Assessment

**Figure 2:**
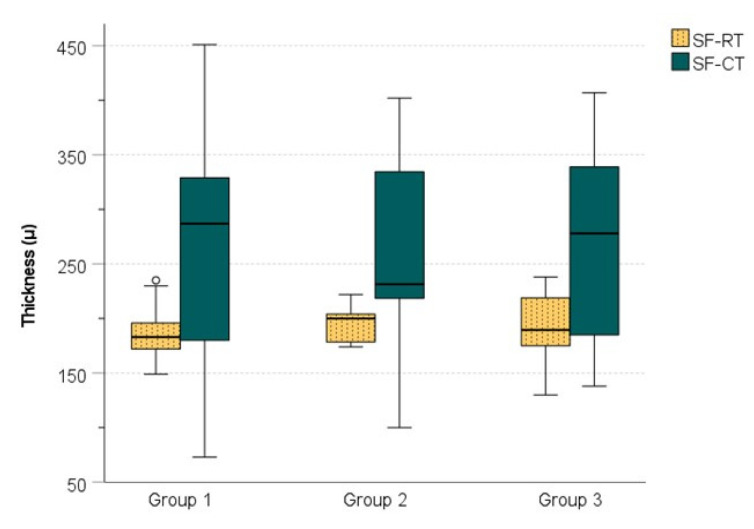
Subfoveal retinal thickness (SF-RT) and subfoveal choroidal thickness (SF-CT) in the three studied groups.

Subfoveal retinal thickness (SF-RT) and subfoveal choroidal thickness (SF-CT) in the three studied groups are presented in Table 2 and Figure 2.

Mean QI was 97.92 ± 2.36 for phakic eyes and 98.46 ± 1.79 for pseudophakic eyes, with no significant differences (*p* = 0.354). In our study, the posterior boundary of the choroid was identified in all subjects. The SCL was present in half of the examined subjects in group 1 and 3 and in 65% examined subjects in group 2, with no significant differences between groups (*p* > 0.05). There were no significant differences between the three groups with respect to SF-RT and SF-CT (*p* > 0.05).

#### 3.2.2. Choroidal Parameters Assessment

1. Reliability assessment

Intra-rater reliability for measured choroidal parameters (TCA and LA) on the binarized images is presented in Table 3. Both for TCA and LA in all three studied areas, ICCs were above 0.95 for single measures and above 0.97 for average measures, indicating a good agreement between the two measurements.

2. Choroidal parameters compared between the studied groups

The mean ± SD and *p* values for choroidal parameters for the three studied areas are presented in Table 4, Table 5 and Table 6.

There were no significant differences in TCA, LA, and SA between the three groups for all studied areas (*p* > 0.05). For the choroidal area under the 1.5 mm horizontal line, both LA/SA-1.5 ratio and CVI-1.5 were significantly lower in group 3 compared to group 1 (*p* = 0.020, and *p* = 0.007, respectively) and group 2 (*p* = 0.022, and *p* = 0,013, respectively). For the choroidal area under the 3 mm horizontal line, only CVI-3 was significantly lower in group 3 compared to group 2 (*p* = 0.01). For the choroidal area under the 6 mm horizontal line, both LA/SA-6 ratio and CVI-6 were significantly lower in group 3 compared to group 2 (*p* = 0.046, and *p* = 0.031, respectively).

3. Choroidal parameters compared between the studied areas

**Figure 3:**
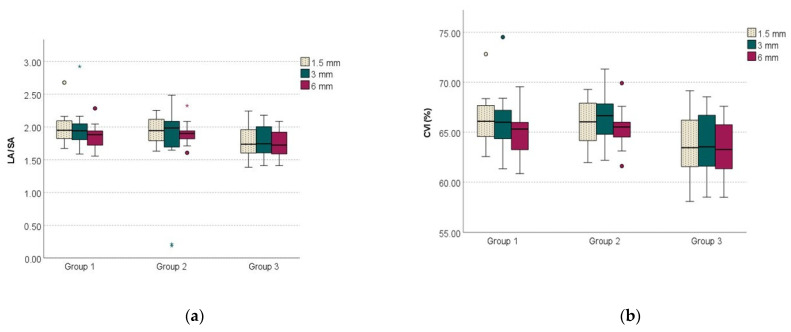
Choroidal parameters calculated as ratios for the three studied areas: (**a**) LA/SA ratio; (**b**) CVI.

When evaluated on the choroidal area under the 3 and 6 mm horizontal line, TCA, LA, and SA significantly increased (*p* < 0.01) in all studied groups, compared to the choroidal area under the 1.5 mm horizontal line. There were no significant differences between the values of the choroidal parameters calculated as ratios (LA/SA and CVI) for the three studied areas (*p* > 0.05) Figure 3.

4. Correlations analysis

We analyzed the correlation of SF-CT, TCA, LA, SA, LA/SA, and CVI with age, SE, axial length, fasting blood sugar, and HbA1c for the three groups.

SF-CT corelated significantly negatively with age in group 1 (*r* = −0.645, *p* = 0.002). In group 2, SF-CT corelated significantly negatively with axial length, fasting blood sugar and HbA1c (*r* = −0.449, *p* = 0.047; *rho* = −0.529, *p* = 0.016, and *rho* = −0.651, *p* = 0.002, respectively) and positively with SE (*rho* = 0.546, *p* = 0.013).

There was a significant negative correlation of TCA-1.5, LA-1.5, and SA-1.5 with age for patients in group 1 (*r* = −0.588, *p* = 0.005; *r* = −0.592, *p* = 0.005, and *r* = −0.572, *p* = 0.007, respectively). With respect to TCA-3, TCA-6, LA-3, LA-6, SA-3, and SA-6, the correlation with age was replicated for group 1.

**Figure 4:**
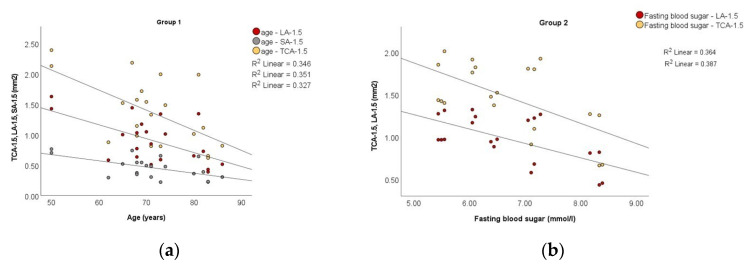

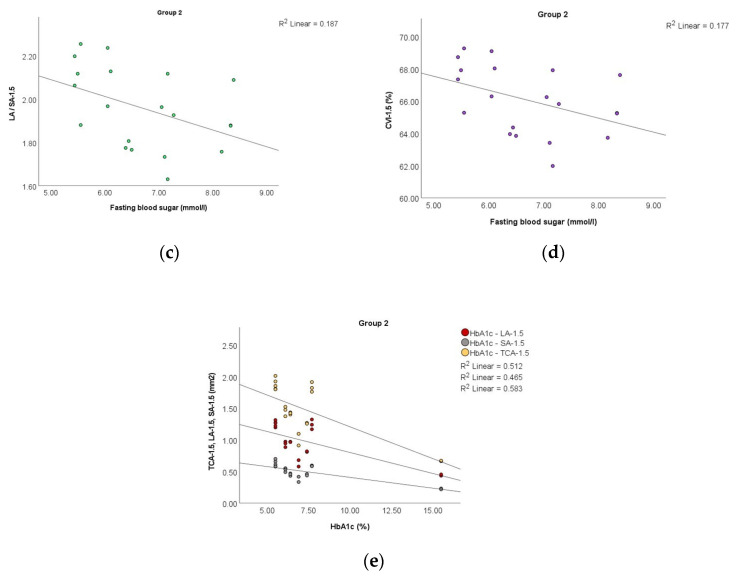
Scatter plots to demonstrate the correlation of: (**a**) TCA-1.5, LA-1.5 and SA-1.5 with age in group 1; (**b**) TCA-1.5, LA-1.5 with fasting blood sugar in group 2; (**c**) LA/SA-1.5 with fasting blood sugar in group 2; (**d**) CVI-1.5 with fasting blood sugar in group 2; (**e**) TCA-1.5, LA-1.5 and SA-1.5 with HbA1c in group 2.

For patients in group 2, TCA-1.5, TCA-6, LA-1.5, LA/SA-1.5, and CVI-1.5 correlated significantly negatively with fasting blood sugar (*rho* = –0.518, *p* = 0.019; *rho* = −0.482, *p* = 0.031; *rho* = −0.565, *p* = 0.009; *rho* = −0.505, *p* = 0.023, and *rho* = −0.505, *p* = 0.023, respectively). In addition, for patients in group 2, TCA-1.5, TCA-3, LA-1.5, LA-3, and SA-1.5 correlated significantly negatively with HbA1c values (*rho* = –0.557, *p* = 0.011; *rho* = −0.592, *p* = 0.006; *rho* = −0.501, *p* = 0.025; *rho* = −0.579, *p* = 0.008, and *rho* = −0.529, *p* = 0.017, respectively), as shown in Figure 4.

For patients in group 3, SF-CT did not correlate with any of the above-mentioned ocular and systemic factors.

The presence of controlled HBP was not associated with SF-CT in any of the three studied groups.

SCL was correlated significantly positively with CVI-3 in group 1 (*r* = 0.481, *p* = 0.027) and group 2 (*r* = 0.437, *p* = 0.042).

Further, we analyzed the association of SF-CT with ocular and systemic factors. Univariable analysis revealed that age, the luminal areas, and the stromal area at the three assessed points were significantly associated with SF-CT in group 1. In the multiple regression model, increasing age and increasing SA-6 remained significantly correlated with a thinner SF-CT.

In group 2, univariable analysis identified a significant association of ocular and systemic factors with SF-CT as follows: SE was positively associated with SF-CT, axial length was negatively associated with SF-CT, fasting blood sugar was negatively associated with SF-CT, HbA1c was negatively associated with SF-CT, SF-RT was positively associated with SF-CT, the luminal areas at the three assessed areas were positively associated with SF-CT, SA-1.5 and SA-6 were positively associated with SF-CT, and CVI-6 was positively associated with SF-CT. However, after multiple regression analysis axial length, fasting blood sugar, HbA1c, and SF-RT were significantly associated factors with SF-CT. Increased axial length and increased fasting blood sugar were significantly associated with decreased SF-CT. Increased HbA1c and SF-RT were associated with a higher SF-CT, as shown in Table 7.

In group 3, univariable analysis identified significant associations of the luminal area, stromal areas, luminal area/stromal area, and CVI in all three assessed areas with SF-CT. After performing multiple regression analyses, there were no significantly associated factors with SF-CT in group 3.

Further, we analyzed the association of CVI with ocular and systemic factors for all three studied areas.

When CVI-1.5 was the dependent variable, univariable analysis identified a significant association of CVI-1.5 with BCVA and IOP in group 2 and with SF-CT, SA-1.5, and SA-3 in group 3. After multiple regression analyses, these factors did not associate with CVI-1.5.

When CVI-3 was the dependent variable, univariable analysis identified a significant association of CVI-3 with IOP in group 2 (*β* = –0.532, *p* = 0.016). An increase in IOP was associated with lower CVI-3. In group 3, univariable analysis identified a significant association of CVI-3 with stromal areas in all three studied areas. After multiple regression analyses, these factors did not associate with CVI-3.

When CVI-6 was the dependent variable, univariable analysis identified a significant association of CVI-6 with SE in group 2 (*β* = 0.443, *p* = 0.050). In group 3, univariable analysis identified a significant association of CVI-6 with SF-CT and SA in all three studied areas. After multiple regression analyses, these factors did not associate with CVI-6 (extended analysis for univariable and multivariable regression analysis is presented in the supplementary material).

## 4. Discussion

The choroid is a complex structure, and the increase in thickness can occur due to vascular, stromal or both components. The heterogeneous structure and the presence of fenestrated blood vessels make it difficult to evaluate the choroid in vitro and in vivo [23].

Of particular importance in the assessment of CT is the correct identification of the posterior limit of the choroid [28,33,34]. In our study, the identification of the CSJ was possible in all the examined subjects, a result similar to those reported in the literature [35].

SCL also has an essential role in the evaluation of CT, and when comparing the results between different studies, one must consider the clear definition of the CSJ and the presence of SCL. Yiu et al. studied the CSJ and the SCL in healthy subjects and concluded that the CT values are different depending on the presence of SCL and how CSJ is defined [24]. In our study, we excluded SCL from the evaluation of choroidal thickness, and the choroid was measured manually. In addition, SCL was not included in the polygonal selection of the ROI when we evaluated the choroidal parameters by binarizing the OCT images. Having a hyporeflective appearance on OCT images, SCL could lead to a false increase in vascular area after image binarization and modify CVI values [34]. After the binarization, if errors were observed at the posterior choroidal boundary, most often by not completely including the choroidal stromal area, the edges of the selection polygon were adjusted to include the choroidal stroma.

Ferrara et al. identified that choroidal vascular remodeling occurs in all patients with DM with or without DR. Outcomes of different studies on CT values in patients with DM and DR are polymorphic without a consensus [30]. Wang et al. reported in a SS-OCT study a thicker choroid in the early stages of DR; as DR progresses, there is a reduction in CT [18]. The authors hypothesized that diabetes may be an independent factor contributing to choroidal thickening. Subsequently, DR progression may lead to choroidal thinning [18]. Laíns et al. reported a significant reduction in CT only in patients with PDR compared to control subjects [36]. In the “Beijing Eye Study”, Xu et al. evaluated SF-CT in patients with DM and in patients with DR and concluded that the former had a significantly higher SF-CT. Moreover, DR and DR stage were not associated with abnormal SF-CT [37]. VEGF-induced vascular dilation, choroidal expansion due to osmotically active molecules, and increased permeability of choroidal vascular endothelial cells, although to a lesser extent compared to retinal vascular endothelial cells [38], are some possible explanations for the determinism of CT growth in patients with diabetes.

In our study, there were no statistically significant differences between the three groups for SF-CT. These results may be due to the small group of patients or due to subtle choroidal changes. Sudhalkar et al. observed a thicker choroid in control subjects compared to patients with DM, with or without DR. The process of choroidal thinning was more severe with the severity of DR, which could contribute to the pathogenesis of DR, explaining the persistence a functional retinal abnormality despite adequate ocular and systemic treatment [39]. Esmaeelpour et al. identified a thinning of the choroid in patients with type 1 diabetes, regardless of the duration of the disease and the presence of DR. Analyzing the two-dimensional topographic maps of CT, they observed that areas of choroidal thinning correspond to retinal lesions, suggesting possible involvement of the choroid in the onset or progression of ocular disease [40]. However, Kim et al. observed an increase in CT with DR severity and a reduction in CT after panretinal photocoagulation [41]. In a systematic review of the recent literature in the PubMed database on choroidal structural alterations in diabetic patients, Hamadneh et al. grouped and classified the studies into different categories, depending on the increase, decrease or absence of changes in CT in patients with DM and according to the progression of DR, and concluded that CT is not a reliable parameter because it correlates poorly with worsening DR or EMD [42]. The discrepancies identified between different studies are explained by the presence of numerous ocular and systemic factors which could contribute to CT changes, such as axial length, SE, IOP, intraocular treatments, age, sex, diurnal variation, duration of DM, DM treatment, and HBP [43].

In our study, OCT image acquisition was performed between 1 p.m. and 3 p.m. to overcome the diurnal variation of the choroid. Previous studies identified that between 12 p.m. and 3 p.m., the choroid has the lesser variation compared to other horary intervals [26,27]. We analyzed the factors that could contribute to CT variation separate for the three groups: non-diabetic patients, diabetic patients without DR, and diabetic patients with NPDR. After multivariable regression analyses, we found that age was associated with a thinner SF-CT only in non-diabetic patients and axial length was associated with a thinner SD-CT only in diabetic patients without DR. In multiple regression analyses, an increase in the SA calculated for a choroidal area under a 6 mm horizontal line was associated with a thinner SF-CT in non-diabetic patients. No associations were found between SF-CT and sex and IOP in univariable analysis.

With respect to DM duration, we found no association with SF-CT. We evaluated glycemic control by fasting blood sugar and HbA1c. In our study, these two factors were associated with SF-CT both in univariable and multivariable analyses in patients with DM without DR. Fasting blood sugar was associated with a thinner SF-CT, and HbA1c was associated with thicker SF-CT. In a study evaluating the influence of glycemic parameters on CT in patients with DM without DR and in patients with DM and DR, Abalem et al. found a positive correlation between capillary blood glucose and CT in patients with NPDR, but not in those with PDR [44]. However, they found no correlation of fasting blood sugar and HbA1c with CT. Torabi et al. investigated the correlation between CT and HbA1c in patients with type 2 diabetes in an SD-OCT study in which both eyes from patients with diabetes were divided into three groups according to HbA1c values ( ≤7% good control, 7–8% moderate control and ≥8% poor control) and concluded that there is a significant correlation of CT with HbA1c in patients with DM, and an HbA1c ≤ 7% may prevent choroidal thinning [45]. In our study, the correlation between CT and HbA1c was analyzed between the groups of patients without establishing cut-off values for HbA1c, which would have led to excessive fragmentation of the groups. In a similar approach to our study with respect to patient grouping according to the stage of DR, Sahinoglu-Keskek et al. found no correlations between SF-CT and HbA1c [46].

In our study, the presence of controlled HBP was not associated with SF-CT in any of the three studied groups. Several studies on healthy subjects versus subjects with HBP mention HBP as a systemic factor associated with a thinner CT [47,48]. The proposed mechanism is vasoconstriction of choroidal vessels induced by HBP. On the contrary, Gök et al. found no differences between healthy subjects and subjects with HBP with respect to SF-CT [49], most likely due to the intrinsic choroidal characteristics that provide relative protection against chronic hypertension [47].

Patients with DM and NPDR did not show associations of SF-CT with any of the above-mentioned ocular and systemic factors.

CT is used as a surrogate biomarker for the evaluation of choroidal vascularization, but it cannot indicate the affected choroidal component [50]. Agrawal et al. defined the ratio between LA and TCA as CVI [25]. CVI is considered a better and stable quantitative parameter and a consistent indirect measure of choroidal vascularity assessment compared to CT, because it is less influenced by other factors [34]. In our study, there were no statistically significant differences between groups with respect to CT, but CVI was found to decrease significantly in diabetic patients with NPDR compared to DM patients for all three studied areas. In animal models, it has been observed that decreased ocular blood flow is an early indicator of retinopathy [51]. It is unclear whether choroidal vascular changes precede or follow DR, but there are studies that have identified reductions in choroidal flow prior to DR, suggesting that choroidal ischemic changes play an important role in DR pathogenesis. Foo et al. reported macular CVI decrease in Haller’s layer, but not in the Sattler layer in patients with DM without DR [52]. Kim et al. retrospectively analyzed CVI in patients with type 2 diabetes versus healthy subjects using SS-OCT and observed that patients with diabetes had a significantly lower CVI compared to control eyes, even in the absence of DR [53]. In our study, we found that CVI was lower in patients with DM and NPDR compared to patients with DM without DR for all three studied areas. No significant differences in CVI values were identified between the non-diabetic patients and those with DM without DR for any of the studied areas. Tan et al. also found a statistically significant reduction in CVI in patients with diabetes compared to the control group. CVI had 81.6% sensitivity and 60.5% specificity in identifying choroidal changes in DM subjects [23].

Regarding TCA, LA, SA, we did not found statistically significant differences in the three studied groups. Tan et al. found no differences in TCA, LA, and SA in patients without DM versus patients with DM with or without DR [23]. Conversely, Damian et al. reported a significant difference between normal subjects and pooled DM and DM with mild NPDR subjects regarding TCA, LA, and SA, but no differences when they evaluated the DM versus DM with mild NPDR subjects [54].

Irrespective of the studied group or the area for which CVI was calculated, we did not find correlations between CVI and SF-CT. Our results are consistent with other reports [23]. In a multivariable analysis, Kim et al. found that a thicker SF-CT and a thinner central retina were significantly associated with higher CVI values [53]. In our study, when we considered CVIs as dependent variables, we identified only a few independent ocular factors to be associated in univariable analysis with CVI in different studied areas. However, these independent factors did not show any association with CVI in multivariable analysis. Moreover, none of the systemic independent factors such as fasting blood sugar, HbA1c, diabetes duration and treatment and the presence of HBP were associated in univariable analysis with CVIs. These findings could prove that CVI, regardless of the area of assessment, is a stable and reliable indicator of choroidal changes in the presence of DM. However, Aşıkgarip et al. found that HBP in treatment-naive patients impacts the SF-CT, LA and CVI and reported a negative correlation between choroidal parameters and HBP [55]. In our study, both SF-CT and CVI were not associated with the presence of HBP. We included only patients without HBP or with controlled HBP. Thus, the independent status of the SF-CT and CVI found in our study relative to HBP is questionable, since the patients had no overt HBP. However, in this study, no difference between patients without HBP and those with controlled HBP was found in regard to SF-CT and CVI. In addition, the cross-sectional design of the study could be a drawback from this point of view as we could not assume that patients with controlled HBP have no choroidal vascular changes induced before treatment initiation. In this respect, we can hypothesize that undiagnosed HBP could induce choroidal alterations before treatment initiation reflected or not thereafter in the choroidal parameters. The level of measurement in our study for this systemic factor wasnominal (yes/no). Further longitudinal studies assessing SF-CT and CVI in several subgroups (no HBP, controlled HBP and uncontrolled HBP) could more accurately address this aspect and a quantitative level of measurement of systemic blood pressure, which permits to calculate the mean and SD could draw a clearer line of the influences of this factor on choroidal assessment.

In DM patients, a process of choroidal remodeling takes place, with vascular narrowing and capillary loss [30], which reduce the vascular proportion and therefore the CVI.

Agrawal et al. reported in a previous study on healthy subjects that the CVI measured from a single foveal scan is representative for total macular CVI [56]. Pathology studies indicate that DC changes are not necessarily diffuse. To overcome the limitation of binarization on a restricted area at the subfoveal level and assuming that the choroidal changes are not necessarily homogeneous and diffuse, even in early DR stages, we evaluated the choroidal parameters on three increasing choroidal areas centered on fovea. The differences in CVI values observed between the studied groups were maintained for all three measurements, which may suggest that the identified choroidal changes are homogeneous and diffuse in the early stages of DR.

Several limitations of the study can be mentioned. Despite aiming to evaluate choroidal changes in patients with NPDR, the authors acknowledge that the small number of patients included in the study prevented an individualized assessment per every stage of NPDR. Further studies need to also clarify whether the CVI is a reliable indicator for various stages of NPDR. In our study, we assessed only SF-CT and searched for the influence of the three choroidal areas upon the subfoveal area. The authors aim to evaluate, in the future, the correlations of different CTs with different choroidal areas. Image binarization itself could be a limitation by assuming that black pixels are vascular areas and white pixels are stromal areas. In our study, we used a method that two-dimensionally evaluates a horizontal section that passes through the center of the fovea. A three-dimensional assessment of the choroid would provide an overview of the choroidal vascular parameters and could demonstrate whether, in the early stages, the changes are homogeneous and diffuse for the entire choroid. Last but not least, monitoring the longitudinal evolution of CVIs might indicate whether measuring this parameter is useful in monitoring DC and DR in patients with diabetes.

To the best of our knowledge, this is the first SS-OCT study to evaluate SCL and exclude it from image processing and measurements and to consider three standardized areas (1.5, 3, and 6 mm) for choroidal assessment and two polygonal selections, before and after binarization process, with fine adjustments of the polygon edges for a better delineation of the posterior choroidal boundary.

## 5. Conclusions

Choroidal assessment by swept-source OCT and image binarization in normal subjects, subjects with DM without DR, and subjects with DM and NPDR without DME indicated that SF-CT, TCA, LA, and SA were similar between groups. Significant CVI changes were present even in early DR compared to DM, but no association with ocular and systemic factors was found, suggesting that CVIs are reliable parameters of choroidal vascular structure.

## Figures and Tables

**Table 1:** Demographical, ocular, and biochemical characteristics in the study groups.

Parameters	Group 1 (*n* = 21)	Group 2 (*n* = 20)	Group 3 (*n* = 22)	*p* ^1^
Age (years)	71.14 ± 9.69	71.75 ± 9.03	63.41 ± 5.84	0.002 *
Sex (*n*, male: female)	9:12	8:12	9:13	0.982 ^^^
BCVA (logMAR)	0.26 ± 0.23	0.14 ± 0.15	0.27 ± 0.22	0.056 ^˘^
SE (D)	0.26 ± 1.50	0.08 ± 1.18	0.16 ± 1.54	0.907 ^˘^
IOP (mmHg)	13.90 ± 3.41	14.70 ± 1.75	16.86 ± 2.23	0.001 *
Lens status (*n*, phakic: pseudophakic)	10:11	9:11	20:2	0.002 ^^^
Axial length (mm)	22.78 ± 0.74	22.76 ± 0.61	22.87 ± 0.72	0.879 *
Fasting blood sugar (mmol/l)	5.63 ± 0.54	6.70 ± 1.02	9.67 ± 4.03	0.001 ^˘^
HbA1c (%)	5.64 ± 0.45	7.38 ± 2.88	7.96 ± 1.78	0.001 ^˘^
Diabetes duration (years)	N/A	7.30 ± 2.69	9.59 ± 6.85	1 ^×^
DM treatment (*n*, OADs: OADs + insulin)	N/A	19:1	13:9	0.01 ^ᵜ^
HBP (*n*, %)	17 (80.95)	17 (85)	18 (77.27)	1 ^ᵜ^

^1^ significance of differences between groups; N/A: non applicable * one-way ANOVA test; ^^^ Pearson Chi-square test; ^˘^ Kruskal–Wallis test; ^×^ Mann–Whitney U test; ^ᵜ^ Fisher exact test; BCVA: best corrected visual acuity; logMAR: logarithm of the minimum angle of resolution; SE: spherical equivalent; D: diopters; IOP: intraocular pressure; HbA1c: glycated hemoglobin; OADs: oral antidiabetic drugs; DM: diabetes mellitus; HBP: high blood pressure.

**Table 2:** Subfoveal retinal and choroidal thickness and presence of suprachoroidal layer.

Parameter	Group 1 (*n* = 21)	Group 2 (*n* = 20)	Group 3 (*n* = 22)	*p* ^1^
SF-RT (μ)	188.67 ± 21.93	195.00 ± 16.57	192.73 ± 28.68	0.401 ^˘^
SF-CT (μ)	260.19 ± 113.18	258.40 ± 85.43	262.64 ± 85.18	0.990 *
SCL (*n*, %)	11 (52.4)	13 (65%)	11 (50)	0.627 ^^^

^1^ significance of differences between groups; ^˘^ Kruskal–Wallis test; * one-way ANOVA test; ^^^ Pearson Chi-square test; SF-RT: subfoveal retinal thickness; SF-CT: subfoveal choroidal thickness; SCL: suprachoroidal layer.

**Table 3:** Intra-rater reliability for measured choroidal parameters.

	Single Measures		Average Measures	
Measured Parameter	ICC	95% CI	ICC	95% CI
TCA-1.5	0.954	0.889–0.977	0.977	0.941–0.989
TCA-3	0.958	0.959–0.988	0.978	0.959–0.988
TCA-6	0.983	0.970–0.990	0.991	0.985–0.995
LA-1.5	0.951	0.859–0.977	0.975	0.924–0.989
LA-3	0.955	0.907–0.976	0.977	0.951–0.988
LA-6	0.974	0.948–0.986	0.987	0.974–0.993

ICC: intra-class correlation coefficient; CI: confidence interval; TCA-1.5: total choroidal area measured for the choroidal area under a 1.5 mm horizontal line centered on the fovea; TCA-3: total choroidal area measured for the choroidal area under a 3 mm horizontal line centered on the fovea; TCA-6: total choroidal area measured for the choroidal area under a 6 mm horizontal line centered on the fovea; LA-1.5: luminal area measured for the choroidal area under a 1.5 mm horizontal line centered on the fovea; LA-3: luminal area measured for the choroidal area under a 3 mm horizontal line centered on the fovea; LA-6: luminal area measured for the choroidal area under a 6 mm horizontal line centered on the fovea.

**Table 4:** Choroidal parameters in the studied groups for the choroidal area under the 1.5 mm horizontal line centered on the fovea.

Choroidal Parameter	Group 1 (*n* = 21)	Group 2 (*n* = 20)	Group 3 (*n* = 22)	*p* ^1^
TCA-1.5 (mm^2^)	1.36 ± 0.54	1.46 ± 0.40	1.41 ± 0.44	0.787 *
LA-1.5 (mm^2^)	0.90 ± 0.37	0.97 ± 0.28	0.91 ± 0.31	0.777 *
SA-1.5 (mm^2^)	0.45 ± 0.17	0.49 ± 0.12	0.50 ± 0.14	0.539 *
LA/SA-1.5	1.97 ± 0.22	1.95 ± 0.18	1.77 ± 0.22	0.007 ^˘^
CVI-1.5 (%)	66.21 ± 2.37	66.06 ± 2.11	63.74 ± 2.97	0.003 *

^1^ significance of differences between groups; * one-way ANOVA test; ^˘^ Kruskal–Wallis test; TCA-1.5: total choroidal area measured for the choroidal area under the 1.5 mm horizontal line centered on the fovea; LA-1.5: luminal area measured for the choroidal area under a 1.5 mm horizontal line centered on the fovea; SA-1.5: stromal area calculated for the choroidal area under a 1.5 mm horizontal line centered on the fovea; CVI-1.5: choroidal vascularity index calculated for the choroidal area under a 1.5 mm horizontal line centered on the fovea.

**Table 5:** Choroidal parameters in the studied groups for the choroidal area under the 3 mm horizontal line centered on the fovea.

Choroidal Parameter	Group 1 (*n* = 21)	Group 2 (*n* = 20)	Group 3 (*n* = 22)	*p* ^1^
TCA-3 (mm^2^)	2.72 ± 1.04	2.81 ± 0.83	2.75 ± 0.84	0.946 *
LA-3 (mm^2^)	1.80 ± 0.71	1.87 ± 0.56	1.77 ± 0.60	0.871 *
SA-3 (mm^2^)	0.92 ± 0.33	1.60 ± 1.97	0.97 ± 0.25	0.115 *
LA/SA-3	1.95 ± 0.27	1.82 ± 0.60	1.78 ± 0.22	0.106 ^˘^
CVI-3 (%)	65.88 ± 2.81	66.46 ± 2.62	63.79 ± 2.94	0.008 *

^1^ significance of differences between groups; * one-way ANOVA test; ^˘^ Kruskal–Wallis test; TCA-3: total choroidal area measured for the choroidal area under the 3 mm horizontal line centered on the fovea; LA-3: luminal area measured for the choroidal area under a 3 mm horizontal line centered on the fovea; SA-3: stromal area calculated for the choroidal area under a 3 mm horizontal line centered on the fovea; CVI-3: choroidal vascularity index calculated for the choroidal area under a 3 mm horizontal line centered on the fovea.

**Table 6:** Choroidal parameters in the studied groups for the choroidal area under the 6 mm horizontal line centered on the fovea.

Choroidal Parameter	Group 1 (*n* = 21)	Group 2 (*n* = 20)	Group 3 (*n* = 22)	*p* ^1^
TCA-6 (mm^2^)	4.91 ± 2.08	5.27 ± 1.28	5.16 ± 1.65	0.791 *
LA-6 (mm^2^)	3.20 ± 1.40	3.45 ± 0.87	3.31 ± 0.18	0.790 *
SA-6 (mm^2^)	1.71 ± 0.67	1.81 ± 0.43	1.85 ± 0.53	0.688 *
LA/SA-6	1.85 ± 0.17	1.89 ± 0.15	1.76 ± 0.20	0.046 ^˘^
CVI-6 (%)	64.79 ± 2.08	65.40 ± 1.76	63.61 ± 2.62	0.032 *

^1^ significance of differences between groups; * one-way ANOVA test; ^˘^ Kruskal–Wallis test; TCA-6: total choroidal area measured for the choroidal area under the 6 mm horizontal line centered on the fovea; LA-6: luminal area measured for the choroidal area under a 6 mm horizontal line centered on the fovea; SA-6: stromal area calculated for the choroidal area under a 6 mm horizontal line centered on the fovea; CVI-6: choroidal vascularity index calculated for the choroidal area under a 6 mm horizontal line centered on the fovea.

**Table 7:** Multiple linear regression analysis of factors associated with subfoveal choroidal thickness.

		Multivariable ^1^			95% CI for B	
Independent Variables	Group	Unstandardized B	Standardized *β*	*p*	Lower Bound	Upper Bound
Age (years)	1	−3.131	−0.268	0.017	−5.600	−0.663
SA-6 (mm^2^)	1	−322.776	−1.937	0.037	−622.885	−22.667
Axial length (mm)	2	−33.940	−0.243	0.001	−49.728	−18.151
Fasting blood sugar (mmol/L)	2	−51.485	−0.618	0.001	−72.085	−30.885
HbA1c	2	3.886	0.131	0.040	0.230	7.543
SF-RT (μ)	2	3.174	0.616	0.001	2.012	4.337

^1^ adjusted for variables with a *p* < 0.05 in the univariable analysis. Only variables with statistical significance are shown (univariable analysis is presented in the Supplementary Material).

## Data Availability

The data published in this research are available on request from the first author (O.O.).

## Supplementary Materials

The following supporting information can be downloaded at: https://www.mdpi.com/article/10.3390/medicina58070918/s1, Table S1: Linear regression analysis of factors associated with subfoveal choroidal thickness for group 1; Table S2: Linear regression analysis of factors associated with subfoveal choroidal thickness for group 2; Table S3: Linear regression analysis of factors associated with subfoveal choroidal thickness for group 3; Table S4: Linear regression analysis of factors associated with CVI-1.5 for group 1; Table S5: Linear regression analysis of factors associated with CVI-1.5 for group 2; Table S6: Linear regression analysis of factors associated with CVI-1.5 for group 3; Table S7: Linear regression analysis of factors associated with CVI-3 for group 1; Table S8: Linear regression analysis of factors associated with CVI-3 for group 2; Table S9: Linear regression analysis of factors associated with CVI-3 for group 3; Table S10: Linear regression analysis of factors associated with CVI-6 for group 1; Table S11: Linear regression analysis of factors associated with CVI-6 for group 2; Table S12: Linear regression analysis of factors associated with CVI-6 for group 3.
