# Supplementary material for: Choroidal Assessment in Patients with Type 2 Diabetes Mellitus and Non-Proliferative Diabetic Retinopathy by Swept-Source Ocular Coherence Tomography and Image Binarization
Source: Medicina (Kaunas). 2022 Jul 10;58(7):918. doi: 10.3390/medicina58070918 (PMC9319764; doi:10.3390/medicina58070918)
Supplement: Supplementary file 1 [file medicina-58-00918-s001.zip › medicina-1786494-Supplementary material.pdf]

Table S1: Linear regression analysis of factors associated with subfoveal choroidal thickness for group 1.

Variables	Univariate			Multivariate[1]		
	Unstandardized B	Standardized β	p	Unstandardized B	Standardized β	p
Age (years)	–7.529	–0.645	0.002	–3.131	–0.268	0.017
Sex, female	3.833	0.017	0.941			
BCVA (logMAR)	–185.583	–0.382	0.087			
SE (D)	–9.367	–0.124	0.591			
IOP (mmHg)	4.890	0.148	0.523			
Lens status, phakic	26.173	0.118	0.609			
Axial length (mm)	–7.686	–0.051	0.828			
Fasting blood sugar (mmol/l)	–8.887	–0.042	0.855			
HbA1c (%)	–54.028	–0.219	0.341			
HBP, present	65.088	0.231	0.313			
SF-RT (μ)	0.206	0.040	0.864			
LA-1.5 (mm^2)	290.333	0.959	0.001	250.669	0.828	0.306
LA-3 (mm^2)	150.630	0.952	0.001	–295.360	–1.867	0.097
LA-6 (mm^2)	75.456	0.938	0.001	156.572	1.946	0.050
SA-1.5 (mm^2)	631.879	0.964	0.001	784.878	1.198	0.082
SA-3 (mm^2)	315.909	0.949	0.001	216.424	0.650	0.345
SA-6 (mm^2)	156.582	0.940	0.001	–322.776	–1.937	0.037
LA/SA-1.5 (mm^2)	120.725	0.239	0.297			
LA/SA-3 (mm^2)	39.125	0.095	0.681			
LA/SA-6 (mm^2)	255.971	0.387	0.083			
CVI-1.5 (mm^2)	15.062	0.316	0.162			
CVI-3 (mm^2)	7.989	0.199	0.388			
CVI-6 (mm^2)	23.474	0.432	0.051			

[1] adjusted for variables with a $p < 0.05$ in the univariate analysis.

Table S2: Linear regression analysis of factors associated with subfoveal choroidal thickness for group 2.

Variables	Univariable			Multivariable[1]		
	Unstandardized B	Standardized β	p	Unstandardized B	Standardized β	p
Age (years)	–1.041	–0.11	0.644			
Sex, female	11.50	0.068	0.777			
BCVA (logMAR)	–193.73	–0.34	0.142			
SE (D)	37.04	0.514	0.020	3.861	0.054	0.337
IOP (mmHg)	13.75	0.282	0.229			
Lens status, phakic	–6.78	–0.041	0.865			
Axial length (mm)	–62.74	–0.449	0.047	–33.940	–0.243	0.001
Fasting blood sugar (mmol/l)	–44.05	–0.529	0.016	–51.485	–0.618	0.001
HbA1c (%)	–20.25	–0.684	0.001	3.886	0.131	0.040
Diabetes duration (years)	–0.423	–0.013	0.955			
DM treatment, OADs	–76.42	–0.2	0.398			
HBP, present	–72.86	–0.312	0.180			
SF-RT (μ)	3.158	0.613	0.004	3.174	0.616	0.001
LA-1.5 (mm^2)	285.727	0.939	0.001	68.826	0.226	0.312
LA-3 (mm^2)	138.304	0.917	0.001	3.519	0.023	0.880
LA-6 (mm^2)	83.756	0.853	0.001	–74.974	–0.764	0.361

SA-1.5 (mm²)	613.289	0.926	0.001	144.120	0.218	0.090
SA-3 (mm²)	–3.766	–0.087	0.715			
SA-6 (mm²)	142.145	0.724	0.001	102.875	0.524	0.528
LA/SA-1.5 (mm²)	139.899	0.301	0.198			
LA/SA-3 (mm²)	34.817	0.245	0.297			
LA/SA-6 (mm²)	307.963	0.546	0.013	–116.156	–0.206	0.603
CVI-1.5 (mm²)	12.394	0.307	0.189			
CVI-3 (mm²)	6.042	0.186	0.433			
CVI-6 (mm²)	25.338	0.522	0.018	1824.955	0.376	0.396

[1] adjusted for variables with a $p < 0.05$ in the univariate analysis.

Table S3: Linear regression analysis of factors associated with subfoveal choroidal thickness for group 3.

Variables	Univariable			Multivariable[1]		
	Unstandardized B	Standardized *β*	*p*	Unstandardized B	Standardized *β*	*p*
Age (years)	–0.626	–0.043	0.850			
Sex, female	19.504	0.115	0.610			
BCVA (logMAR)	–193.738	–0.340	0.142			
SE (D)	8.212	0.148	0.510			
IOP (mmHg)	6.013	0.158	0.484			
Lens status, phakic	–35.600	–0.123	0.586			
Axial length (mm)	–6.698	–0.057	0.800			
Fasting blood sugar (mmol/l)	–0.640	–0.030	0.894			
HbA1c (%)	6.788	0.142	0.528			
Diabetes duration (years)	–1.799	–0.145	0.521			
DM treatment, OADs	–5.692	–0.034	0.882			
HBP, present	–61.556	–0.285	0.198			
SF-RT (μ)	0.069	0.023	0.919			
LA-1.5 (mm²)	183.715	0.673	0.000	–1572.942	–5.765	0.525
LA-3 (mm²)	96.075	0.677	0.001	1854.476	13.069	0.393
LA-6 (mm²)	49.173	0.656	0.001	–213.472	–2.850	0.777
SA-1.5 (mm²)	386.876	0.652	0.001	2806.650	4.732	0.524
SA-3 (mm²)	223.001	0.665	0.001	–2953.182	–8.811	0.452
SA-6 (mm²)	97.209	0.611	0.002	265.000	1.667	0.841
LA/SA-1.5 (mm²)	161.236	0.432	0.044	–1785.517	–4.789	0.528
LA/SA-3 (mm²)	189.933	0.506	0.016	–2807.192	–7.485	0.649
LA/SA-6 (mm²)	199.137	0.469	0.028	2571.611	6.061	0.361
CVI-1.5 (mm²)	13.311	0.465	0.029	173.825	6.078	0.380
CVI-3 (mm²)	15.360	0.531	0.011	131.575	4.550	0.713
CVI-6 (mm²)	15.540	0.478	0.024	–193.022	–5.942	0.239

[1] adjusted for variables with a $p < 0.05$ in the univariate analysis.

Table S4: Linear regression analysis of factors associated with CVI-1.5 for group 1.

Variables	Univariable			Multivariable[1]		
	Unstandardized B	Standardized *β*	*p*	Unstandardized B	Standardized *β*	*p*
Age (years)	–0.078	–0.318	0.159			
Sex, female	0.291	0.062	0.789			
BCVA (logMAR)	0.786	0.077	0.740			
SE (D)	–0.119	–0.075	0.745			
IOP (mmHg)	–0.064	–0.091	0.693			

Lens status, phakic	0.668	0.144	0.534			
Axial length (mm)	0.406	0.127	0.583			
Fasting blood sugar (mmol/l)	–0.223	–0.051	0.827			
HbA1c (%)	–0.277	–0.053	0.818			
HBP, present	1.359	0.230	0.316			
SF-RT (μ)	0.015	0.142	0.539			
SF-CT (μ)	0.007	0.316	0.162			
SA-1.5 (mm^2)	3.365	0.244	0.286			
SA-3 (mm^2)	1.455	0.208	0.366			
SA-6 (mm^2)	1.021	0.292	0.199			

[1] adjusted for variables with a $p < 0.05$ in the univariate analysis.

Table S5: Linear regression analysis of factors associated with CVI-1.5 for group 2.

Variables	Univariable			Multivariable[1]		
	Unstandardized B	Standardized *β*	*p*	Unstandardized B	Standardized *β*	*p*
Age (years)	–0.017	–0.073	0.759			
Sex, female	–1.001	–0.238	0.312			
BCVA (logMAR)	8.800	0.625	0.003	0.000	0.000	0.999
SE (D)	–0.664	–0.372	0.106			
IOP (mmHg)	–0.545	–0.451	0.046	–0.010	–0.008	0.640
Lens status, phakic	–0.345	–0.083	0.727			
Axial length (mm)	–0.600	–0.174	0.464			
Fasting blood sugar (mmol/l)	–0.867	–0.421	0.065			
HbA1c (%)	0.035	0.048	0.841			
Diabetes duration (years)	–0.036	–0.046	0.848			
DM treatment, OADs	–0.238	–0.025	0.916			
HBP, present	–2.045	–0.355	0.125			
SF-RT (μ)	–0.008	–0.061	0.799			
SF-CT (μ)	0.008	0.307	0.189			
SA-1.5 (mm^2)	2.665	0.163	0.493			
SA-3 (mm^2)	–0.389	–0.363	0.115			
SA-6 (mm^2)	2.117	0.436	0.055			

[1] adjusted for variables with a $p < 0.05$ in the univariate analysis.

Table S6: Linear regression analysis of factors associated with CVI-1.5 for group 3.

Variables	Univariable Unstandardized B	Univariable Standardized *β*	Univariable *p*	Multivariable[1] Unstandardized B	Multivariable[1] Standardized *β*	Multivariable[1] *p*
Age (years)	–0.026	–0.052	0.820			
Sex, female	–0.023	–0.004	0.986			
BCVA (logMAR)	–2.556	–0.189	0.398			
SE (D)	–0.492	–0.254	0.254			
IOP (mmHg)	–0.394	–0.295	0.182			
Lens status, phakic	–3.611	–0.357	0.103			
Axial length (mm)	0.464	0.113	0.615			
Fasting blood sugar (mmol/l)	–0.085	–0.115	0.611			
HbA1c (%)	–0.003	–0.002	0.994			
Diabetes duration (years)	–0.092	–0.211	0.346			
DM treatment, OADs	0.596	0.101	0.656			
HBP, present	–2.345	–0.311	0.159			
SF-RT (μ)	0.036	0.351	0.109			
SF-CT (μ)	0.016	0.465	0.029	0.002	0.052	0.074
SA-1.5 (mm^2)	8.829	0.426	0.048	1.656	0.080	0.372
SA-3 (mm^2)	5.941	0.507	0.016	–0.904	–0.077	0.376
SA-6 (mm^2)	2.276	0.409	0.058			

[1] adjusted for variables with a $p < 0.05$ in the univariate analysis.

Table S7: Linear regression analysis of factors associated with CVI-3 for group 1.

Variables	Univariable Unstandardized B	Univariable Standardized *β*	Univariable *p*	Multivariable[1] Unstandardized B	Multivariable[1] Standardized *β*	Multivariable[1] *p*
Age (years)	–0.105	–0.361	0.118			
Sex, female	0.753	0.136	0.557			
BCVA (logMAR)	1.288	0.107	0.645			
SE (D)	–0.164	–0.088	0.706			
IOP (mmHg)	–0.128	–0.155	0.502			
Lens status, phakic	1.340	0.244	0.287			
Axial length (mm)	0.168	0.044	0.848			
Fasting blood sugar (mmol/l)	–0.158	–0.030	0.896			
HbA1c (%)	–0.262	–0.043	0.854			
HBP, present	1.703	0.243	0.288			
SF-RT (μ)	0.005	0.039	0.866			
SF-CT (μ)	0.005	0.199	0.388			
SA-1.5 (mm^2)	0.1946	0.119	0.606			
SA-3 (mm^2)	0.496	0.060	0.797			
SA-6 (mm^2)	0.797	0.192	0.404			

[1] adjusted for variables with a $p < 0.05$ in the univariate analysis.

Table S8: Linear regression analysis of factors associated with CVI-3 for group 2.

Variables	Univariable			Multivariable[1]		
	Unstandardized B	Standardized *β*	*p*	Unstandardized B	Standardized *β*	*p*
Age (years)	–0.085	–0.293	0.198			
Sex, female	0.265	0.512	0.832			
BCVA (logMAR)	6.442	0.368	0.111			
SE (D)	–0.163	–0.074	0.758			
IOP (mmHg)	–0.799	–0.532	0.016			
Lens status, phakic	0.772	0.150	0.528			
Axial length (mm)	–1.427	–0.332	0.152			
Fasting blood sugar (mmol/l)	–0.116	–0.045	0.850			
HbA1c (%)	0.297	0.326	0.161			
Diabetes duration (years)	–0.036	–0.046	0.848			
DM treatment, OADs	0.148	0.150	0.528			
HBP, present	–0.629	–0.088	0.713			
SF-RT (μ)	0.009	0.056	0.815			
SF-CT (μ)	0.006	0.186	0.433			
SA-1.5 (mm^2)	0.200	0.010	0.967			
SA-3 (mm^2)	–0.025	–0.019	0.937			
SA-6 (mm^2)	–0.439	–0.073	0.761			

[1] adjusted for variables with a $p < 0.05$ in the univariate analysis.

Table S9: Linear regression analysis of factors associated with CVI-3 for group 3.

Variables	Univariable			Multivariable[1]		
	Unstandardized B	Standardized *β*	*p*	Unstandardized B	Standardized *β*	*p*
Age (years)	0.012	0.023	0.919			
Sex, female	0.040	0.007	0.976			
BCVA (logMAR)	–1.541	–0.116	0.609			
SE (D)	–0.174	–0.091	0.687			
IOP (mmHg)	–0.340	–0.258	0.247			
Lens status, phakic	–3.252	–0.325	0.140			
Axial length (mm)	0.749	0.185	0.410			
Fasting blood sugar (mmol/l)	–0.051	–0.070	0.756			
HbA1c (%)	0.051	0.031	0.891			
Diabetes duration (years)	–0.092	–0.211	0.346			
DM treatment, OADs	–0.087	–0.203	0.364			
HBP, present	–2.002	–0.268	0.227			
SF-RT (μ)	0.026	0.256	0.251			
SF-CT (μ)	0.018	0.531	0.011	0.001	0.028	0.256
SA-1.5 (mm^2)	11.636	0.567	0.006	0.305	0.015	0.848
SA-3 (mm^2)	7.010	0.605	0.003	0.235	0.020	0.857
SA-6 (mm^2)	2.779	0.505	0.016	–0.116	–0.021	0.780

[1] adjusted for variables with a $p < 0.05$ in the univariate analysis.

Table S10: Linear regression analysis of factors associated with CVI-6 for group 1.

Variables	Univariable			Multivariable[1]		
	Unstandardized B	Standardized *β*	*p*	Unstandardized B	Standardized *β*	*p*
Age (years)	–0.062	–0.287	0.207			
Sex, female	–0.022	–0.005	0.982			
BCVA (logMAR)	–0.421	–0.047	0.839			
SE (D)	–0.153	–0.110	0.634			
IOP (mmHg)	–0.059	–0.097	0.675			
Lens status, phakic	0.867	0.213	0.354			
Axial length (mm)	0.534	0.191	0.407			
Fasting blood sugar (mmol/l)	–0.392	–0.102	0.660			
HbA1c (%)	–0.234	–0.052	0.824			
HBP, present	1.121	0.217	0.345			
SF-RT (μ)	0.002	0.024	0.971			
SF-CT (μ)	0.008	0.432	0.051			
SA-1.5 (mm^2)	4.243	0.352	0.118			
SA-3 (mm^2)	1.899	0.310	0.171			
SA-6 (mm^2)	1.168	0.381	0.088			

[1] adjusted for variables with a $p < 0.05$ in the univariate analysis.

Table S11: Linear regression analysis of factors associated with CVI-6 for group 2.

Variables	Univariable			Multivariable[1]		
	Unstandardized B	Standardized *β*	*p*	Unstandardized B	Standardized *β*	*p*
Age (years)	–0.079	–0.408	0.074			
Sex, female	0.314	0.090	0.707			
BCVA (logMAR)	–1.118	–0.095	0.689			
SE (D)	0.658	0.443	0.050			
IOP (mmHg)	–0.086	–0.086	0.720			
Lens status, phakic	1.065	0.309	0.185			
Axial length (mm)	–0.861	–0.299	0.200			
Fasting blood sugar (mmol/l)	–0.359	–0.209	0.377			
HbA1c (%)	–0.118	–0.193	0.415			
Diabetes duration (years)	–0.043	–0.065	0.784			
DM treatment, OADs	–0.298	–0.038	0.874			
HBP, present	0.451	0.094	0.694			
SF-RT (μ)	0.040	0.377	0.102			
SF-CT (μ)	0.011	0.522	0.018			
SA-1.5 (mm^2)	5.005	0.367	0.112			
SA-3 (mm^2)	–0.003	–0.003	0.991			
SA-6 (mm^2)	0.046	0.011	0.962			

[1] adjusted for variables with a $p < 0.05$ in the univariate analysis.

Table S12: Linear regression analysis of factors associated with CVI-6 for group 3.

Variables	Univariable			Multivariable[1]		
	Unstandardized B	Standardized *β*	*p*	Unstandardized B	Standardized *β*	*p*
Age (years)	0.013	0.028	0.900			
Sex, female	–0.518	–0.099	0.660			
BCVA (logMAR)	–0.501	–0.042	0.852			
SE (D)	–0.024	–0.014	0.951			
IOP (mmHg)	–0.231	–0.196	0.381			
Lens status, phakic	–2.752	–0.309	0.162			
Axial length (mm)	0.875	0.243	0.276			
Fasting blood sugar (mmol/l)	–0.001	–0.002	0.994			
HbA1c (%)	0.051	0.034	0.879			
Diabetes duration (years)	–0.085	–0.221	0.323			
DM treatment, OADs	0.715	0.137	0.542			
HBP, present	–1.873	–0.282	0.204			
SF-RT (μ)	0.015	0.167	0.458			
SF-CT (μ)	0.015	0.478	0.024	0.00002629	0.001	0.970
SA-1.5 (mm^2)	9.547	0.523	0.013	0.626	0.034	0.643
SA-3 (mm^2)	5.748	0.557	0.007	0.556	0.054	0.615
SA-6 (mm^2)	2.073	0.424	0.049	–0.295	–0.060	0.406

[1] adjusted for variables with a $p < 0.05$ in the univariate analysis.
